# Metabolic aspects of low carbohydrate diets and exercise

**DOI:** 10.1186/1743-7075-1-7

**Published:** 2004-09-30

**Authors:** Sandra J Peters, Paul J LeBlanc

**Affiliations:** 1 Faculty of Applied Health Sciences, Brock University, St. Catharines, ON, Canada L2S 3A1

## Abstract

Following a low carbohydrate diet, there is a shift towards more fat and less carbohydrate oxidation to provide energy to skeletal muscle, both at rest and during exercise. This review summarizes recent work on human skeletal muscle carbohydrate and fat metabolic adaptations to a low carbohydrate diet, focusing mainly on pyruvate dehydrogenase and pyruvate dehydrogenase kinase, and how these changes relate to the capacity for carbohydrate oxidation during exercise.

## Review

Exercise, an acute bout of muscular activity, requires an expenditure of energy above resting levels. This required mechanical energy is provided through the conversion of metabolic fuels into ATP, the base currency of chemical energy. Once produced, ATP is the only direct form of energy that is transferred and utilized by the contractile apparatus within the muscle. Fats are the predominant fuel source of resting skeletal muscle and during exercise, there is a complex interaction between skeletal muscle fat and carbohydrate (CHO) metabolism (see [1] for review). When evaluating the effects of exercise on skeletal muscle fuel utilization, there are many facets that must be taken into consideration. These include intensity and duration of the bout of exercise and the training status of the subjects. During low intensity physical activity (25% maximal oxygen uptake (VO_2max_)), fat supplies the majority of metabolic fuel to exercising skeletal muscle [2]. As physical activity increases to moderate levels (65–70% VO_2max_), there is a shift to more reliance on CHO, specifically muscle glycogen [2]. However, at this level of physical activity, fat oxidation becomes increasingly important as the duration of exercise increases [2] or as training status improves [3]. The studies presented in this review utilize moderately active subjects (maximal oxygen uptake, 50–60 ml·kg^-1^·min^-1^) exercising at a workload of 65–75% VO_2max _for 30–48 min.

The sources of chemical energy that fuel exercising skeletal muscle are available through endogenous depots (intramuscular glycogen and triglycerides) or exogenous sources (plasma glucose and free fatty acids). In turn, these exogenous and endogenous fuel sources are replenished through dietary intake. As a result, there is an important relationship between diet and fuel metabolism in skeletal muscle.

Diets low in carbohydrate content have become increasingly popular as a method of weight loss. These diets that limit daily dietary carbohydrate intake are termed low-carbohydrate diets (LCD). When evaluating the effects of LCD, there are a couple of factors that must be considered, as they may influence the measured outcome. These include the composition of the diet (since a LCD may replace the missing CHOs with either protein or fat), and the duration of the dietary period. For the purpose of this review, LCD will refer primarily to high-fat low-carbohydrate isocaloric diets with <50 g of CHO per day, with a composition of 3–8% CHO, 22–46% protein, and 51–75% fat, and consumed for 3–6 days.

The present paper will briefly review human skeletal muscle metabolism during exercise and the importance of dietary CHO for metabolic energy production. It has been well documented that diets low in carbohydrates result in several metabolic and hormonal adaptations that improve fat oxidation and promote glycogen sparing in exercising skeletal muscle (see [4] for review). However, the mechanism(s) responsible for these changes in exercising skeletal muscle are still debatable, but could be the result of up-regulated fat and/or down-regulated carbohydrate metabolism. The emphasis of the present paper is on adaptive skeletal muscle CHO and fat metabolism in humans, and will compare recent studies that examine the effects of altered diets on key enzymes and how fatty acid composition and re-feeding of carbohydrates following these altered diets affect these enzymes. Data from other mammals are cited where necessary.

### Regulation of carbohydrate oxidation by low-carbohydrate diet

#### Role of pyruvate dehydrogenase

**Figure 1:**
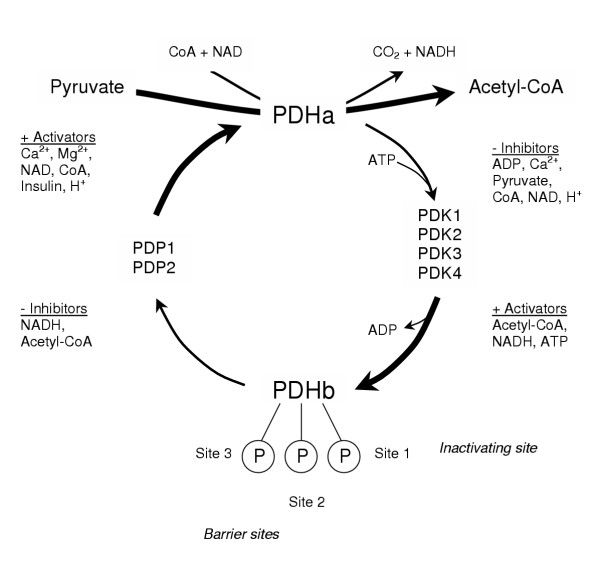
Activation of pyruvate dehydrogenase enzyme complex control by a phosphorylation and dephosphorylation cycle.

In order to understand the regulation of carbohydrate oxidation, the regulation of the mitochondrial enzyme pyruvate dehydrogenase (PDH) must be carefully considered. PDH is a multi-enzyme complex which catalyzes the irreversible oxidative decarboxylation of glycolytically-derived pyruvate to acetyl-coenzyme A (acetyl-CoA; Fig. 1) Because it is highly regulated, it plays a pivotal role in determining the proportion of acetyl-CoA which is derived from carbohydrate sources, thereby regulating flux through carbohydrate oxidation and indirectly determining the rate of fat oxidation. The amount of PDH in its active form (PDHa) determines its activity and regulation is achieved through reversible phosphorylation, catalyzed by an intrinsic PDH phosphatase (PDP), which dephosphorylates and activates PDH, and PDH kinase (PDK), which phosphorylates and inhibits PDH [5]. The E1 subunit of PDH has three known phosphorylation sites, with the first site being necessary for inactivation of the complex, and the other two sites acting as barrier sites to hinder phosphatase activation [6].

Each of the covalent regulatory enzymes of PDH is subject to allosteric regulation. Phosphorylation of the complex is catalyzed by a family of four PDK isoforms (PDK1-4) which differ in their responsiveness to allosteric inhibition by pyruvate or activation by energy charge (ATP/ADP ratio), redox (NADH/NAD^+ ^ratio), and acetyl-CoA-to-free CoA ratio (see [7] for review). In addition, the kinases differ in their specificity for the different phosphorylation sites [7]. Thus, the relative activities of the PDK isoform population will determine the response of the PDH complex in acute situations. An intrinsic pair of phosphatases (PDP1 and 2) catalyze the dephosphorylation and activation of PDH [8]. PDP1 is the isoform which is activated in the presence of increasing concentrations of Ca^2+ ^ions (as would be expected during exercise), while PDP2 is activated when insulin levels are increased during dietary manipulations [8].

At rest, PDH is mainly phosphorylated and inactive due to high energy charge, redox, and acetyl-CoA-to-free CoA ratio and low pyruvate concentration, which maintain a high PDK activity. Phosphatase activity is low at rest, due to low intramuscular Ca^2+ ^levels. During exercise, Ca^2+ ^release from the sarcoplasmic reticulum is the primary stimulus that coarsely activates PDH whereas changes to pyruvate concentration, energy charge, and possibly redox fine-tune this activation (see [9] for review), in order to match PDH activation to the demand for CHO oxidation [10].

In addition to the importance of intramitochondrial effectors to the acute regulation of PDH activation in the first few seconds or minutes, long-term or chronic alterations to the activation state of PDH can be accomplished through stable changes in the absolute levels of PDK and/or PDP. The rate of activation of PDH is dependent on the activity ratio of PDK and PDP, and changes in the expression of either covalent modifier would alter the rate of activation or inactivation of PDH. These chronic alterations occur over hours or days and are independent of acute changes in intramitochondrial effector concentrations.

#### Effects of low-carbohydrate diet

**Figure 2:**
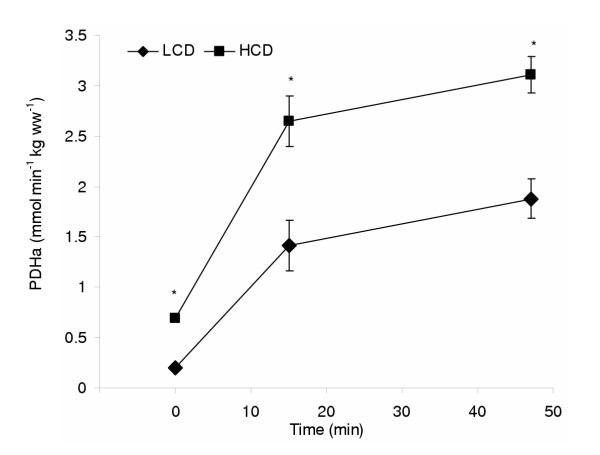
Skeletal muscle pyruvate dehydrogenase in its active form (PDHa) at rest and during exercise in low carbohydrate (LCD) and high carbohydrate (HCD) diets. * denotes significance from LCD. Adapted from Putman et al. [11].

In 1993, Putman and co-workers undertook a study to examine the effects of a short term low-carbohydrate diet on activation of skeletal muscle PDHa activity during moderately intense exercise (75% VO_2max_) [11]. In this study, a 6 d LCD was compared to a high-carbohydrate diet, shifting reliance from the two extremes, either towards fat or towards carbohydrate oxidation. Subjects completed muscle glycogen depleting exercise and then consumed either a LCD (< 3% energy from carbohydrate) or a high-carbohydrate diet (86% carbohydrate) for 6 d. At the end of the dietary intervention, subjects exercised at 75% VO_2max_. The subjects exhausted in ~47 min following the LCD, and exhaustion coincided with hypoglycemia (~2.4 mM) and low levels of muscle glycogen (~32 mmol glucosyl units/kg dry muscle), indicating that that skeletal muscle and liver glycogen stores were limiting under these conditions and at this intensity of exercise. Following the high-carbohydrate diet, exercise was terminated at the same time as the LCD trial, and their blood glucose concentrations were maintained at ~5 mM throughout the exercise duration. Skeletal muscle glycogen content decreased during exercise but was still ~250 mmol glucosyl units/kg dry muscle at the end of exercise. At the onset of exercise during the high-carbohydrate trial, PDHa activity increased maximally in the first 15 minutes of exercise, reflecting the increased energy demand for carbohydrate oxidation at this workload. However, following the LCD, PDHa activity was maximally activated in the first 15 minutes of exercise, but the activation did not achieve the same levels as during the high-carbohydrate trial, effectively impairing the capacity for carbohydrate oxidation and possibly promoting fat oxidation for the duration of exercise at this workload (Fig. 2). The authors were unable to adequately explain the difference in PDHa activity between the trials based on acute changes in the concentrations of intra-mitochondrial effectors, suggesting that chronic regulation of the complex could be playing a role.

**Figure 3:**
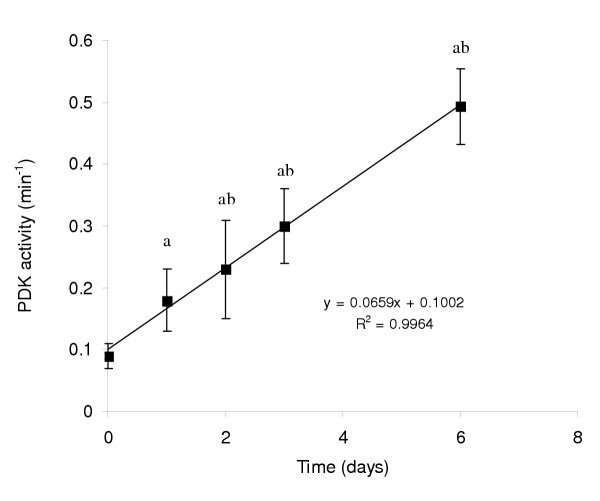
Pyruvate dehydrogenase kinase (PDK) activity during six days of a LCD. ^a ^Significantly different from day 0. ^b ^Significantly different from day 1. Adapted from Peters et al. [12,13].

Subsequent studies demonstrated adaptive alterations at the level of PDK with resultant changes in PDH activation. PDK activity was adaptively increased in human skeletal muscle following 6 days of a LCD [12] (Fig. 3). PDK activity increased in as little as 24 hr and continued to increase in a linear fashion throughout the 6 d diet [13]. The increased PDK activity in human skeletal muscle was associated with increased PDK4 mRNA and protein expression, which was maximally increased after 24 h [13]. These studies suggest a selective increase in PDK4 expression with LCD. The increase in PDK activity during the LCD was associated with impaired glucose clearance from the blood in response to an oral glucose load in health young men [14]. Following as little as 56 h on the LCD, the 90 min area under the blood glucose and plasma insulin concentration vs. time curves increased 2-fold and 1.25-fold, respectively, during an oral glucose tolerance test [14].

These increased levels of PDK4 protein and PDK activity would be expected to render the complex resistant to activation during exercise, as observed by Putman et al. [11] for two reasons: 1) increased multi-site phosphorylation of the PDH complex, and/or 2) decreased sensitivity of the complex to regulation by pyruvate. Increased PDK activity would be expected to enhance multi-site phosphorylation of the E1 subunit and make the complex more resistant to dephosphorylation and activation by the phosphatase [6]. Under normal dietary conditions the predominant isoform in human skeletal muscle is PDK2, which has a greater affinity for phosphorylation of site 1 (the inactivating site) of the E1 subunit [15-17]. However, as the population of PDK4 isoform increased, there would be enhanced phosphorylation of the 2^nd ^(barrier) site, since this isoform has a greater affinity for both site 1 and site 2 [15-17]. As well, PDK2 has a greater sensitivity to inactivation by pyruvate than PDK4 [18]. Thus at the onset of exercise with increased glycolytic flux, the increased levels of PDK4 protein would render the complex more resistant to activation due to increased PDK4 kinase activity even in the face of elevated muscle pyruvate concentrations [19].

A confounding factor in the Putman study was that subjects had undergone intense glycogen depleting exercise protocols prior to both dietary interventions, so the initial levels of skeletal muscle glycogen and glycogen utilization was considerably lower following the LCD [11]. In a subsequent study, subjects were asked to refrain from intense exercise throughout the study, and a LCD (~3% carbohydrate) was compared to a mixed diet (~55–60% carbohydrate) instead of a high-carbohydrate diet [20]. Subjects followed each 6 d dietary intervention with 30 min exercise at a slightly lower workload (65% VO_2max_). The object of the study was to match as closely as possible the glycogen utilization during exercise between the two trials. Although the initial skeletal muscle glycogen concentration was still ~50% lower in the LCD compared to the mixed diet, skeletal muscle glycogen utilization and pyruvate accumulation were similar during the 30 min of exercise in both trials. Unlike the attenuated activation of PDHa at the onset of exercise which was observed in the Putman study [11], these authors observed that the activation during exercise was identical between the two conditions. Thus, in spite of the fact that PDK activity and PDK4 isoform would be expected to increase to a similar extent as previous studies [13], these effects were overridden when initial muscle glycogen levels were higher and glycolytic flux to pyruvate was maintained [20]. It is clear from these studies that the intensity and duration of the exercise play a role in the regulatory changes observed during exercise following a LCD. As exercise intensity increases, the demand for muscle and liver glycogenolysis and muscle carbohydrate oxidation increases. These stores are not fully replenished following a very low carbohydrate diet, and therefore during intense exercise glycogenolytic flux and PDH activation are decreased following a LCD.

#### Effect of fatty acid composition of low-carbohydrate diet

**Figure 4:**
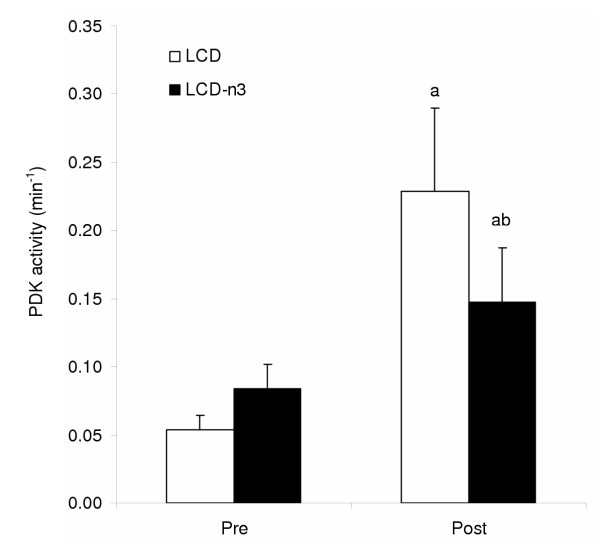
Pyruvate dehydrogenase kinase (PDK) activity before and after three days of a LCD with and without n3 fatty acids. ^a ^Significantly different from pre diet. ^b ^Significantly different from post LCD diet. Adapted from Turvey et al. [21].

The studies presented in this review demonstrate that LCDs decrease the activation of PDH in skeletal muscle at rest and during exercise, mediated through increased PDK activity and isoform expression. However, not all LCDs are created equally, and there is increasing interest in the composition of the fatty acids consumed. Recently, it has been demonstrated that substituting only ~12% of the fat in a LCD (~3% carbohydrate; 75% fat) with fish oils, which are high in omega-3 unsaturated fatty acids, attenuated the diet-induced increase in PDK activity in human skeletal muscle [21] (Fig. 4). These results are similar to earlier work in rodents, with the key difference being that the diet-induced increase in rat skeletal muscle PDK activity was completely abolished with the addition of fish oil [22]. In fact, the increase in PDK activity following a 28 d LCD diet could be completely reversed in as little as 24 h when fish oils were introduced into the high-fat diet [22]. Surprisingly, in both rat and human skeletal muscle in the resting and basal state, PDHa activity was not affected by the inclusion of fish oils suggesting that the total fat content of the diets was more important in determining the conversion of the complex in the basal state [22]. However, there is evidence from animal studies that a LCD which is rich in fish oils enhances muscle carbohydrate oxidation and glucose disposal in response to a challenge such as an insulinemic/euglycemic clamp. Jucker et al. [23] fed rats one of three experimental diets to study the effects on muscle metabolism: a LCD diet rich in safflower oil; a LCD rich in fish oil; or a high-carbohydrate (control) diet. They found that the safflower-fed rats were insulin resistant compared to control or the fish oil-fed rats. The increased whole body glucose disposal in the fish oil-fed rats correlated with increased insulin-stimulated muscle disposal of glucose through oxidation as determined with stable isotope tracer technology [23]. Thus, it would appear that the deleterious effects of high-fat feeding on carbohydrate oxidation and glucose disposal may be ameliorated when the dietary composition of fatty acids are considered carefully. The effect of altered fat composition on skeletal muscle metabolism during exercise has yet to be examined.

#### Effect of re-feeding of carbohydrate following low-carbohydrate diet

In humans, there is little information on how rapidly the LCD-adapted increase in PDK activity and PDK4 protein may be reversed with carbohydrate re-feeding. Most re-feeding studies have used prolonged fasting as a perturbation, and very little work has been done in human skeletal muscle. In rodents, early studies in cardiac muscle indicated that re-feeding following 6 h starvation recovered PDHa activity to ~75% of normal levels in as little as 1–2 h. However, as the duration of the starvation period increased, the time course of the response to re-feeding was longer, in such that after 48 h of starvation, PDHa activity recovered to only ~25% of control values after 4 h [24]. In later rodent studies, this increasing resistance to PDH complex activation was accompanied by increased PDK activity, which correlated with the duration of the fast or high-fat diet [25,26]. Following 48 h starvation and re-feeding, PDK activity and PDK4 protein in skeletal muscle decreased ~50–60% in approximately 4 h of re-feeding [27]. However, little is known about the time course reversion of PDK activity and PDK isoform expression following a LCD in human skeletal muscle.

In human skeletal muscle, Pilegaard et al. [28] recently examined changes in PDK4 mRNA concentrations in human skeletal muscle following fasting and re-feeding. Subjects fasted for 20 h and then were given either a high-carbohydrate meal or a high-fat meal. In muscle biopsies taken 1 h after the re-feeding meal, these authors found increased transcription rate and mRNA concentration of the PDK4 isoform regardless of the composition of the meal. Based on rodent studies of PDK activity, these data were unexpected, since it would be expected that the high-carbohydrate meal would suppress PDK4 gene expression. These data suggested that the skeletal muscle PDK 4 gene was very sensitive to metabolic disturbances. However, without measurement of PDK or PDH activity, the study gave little information regarding how quickly the fasting-induced increase in PDK activity was reversed with carbohydrate re-feeding. A recent study examining carbohydrate re-feeding following a 6 d LCD indicates that PDK activity is rapidly reversed and PDHa activity has fully recovered in as little as 3 h in resting human skeletal muscle [29]. Thus, the adaptive change in PDK activity observed in human skeletal muscle is rapidly reversed with re-feeding of carbohydrates, regardless of potential changes in PDK4 mRNA expression [28].

### Regulation of fat oxidation by low-carbohydrate diet

There is little information regarding the skeletal muscle adaptation on "the fat side" to a LCD. In human skeletal muscle, most studies restrict their measurements to gene expression or mRNA concentrations of the pertinent enzymes involved in fat oxidation, and very few have measured the more physiologically relevant concentrations of enzyme activity or protein concentration. Still, there is evidence in human skeletal muscle for increased activities of several regulatory enzymes and proteins in skeletal muscle fatty acid uptake and oxidation following high fat diets or LCD. Key steps include delivery of fatty acids to the muscle through muscle lipoprotein lipase (LPL), sarcolemmal fatty acid transporters and plasma membrane fatty acid binding proteins (FAT/CD26 and FABP_pm _respectively), mitochondrial uptake and oxidation through carnitine palmitoyl transferase I (CPT I), fatty acid beta-oxidation (marker enzyme β-hydroxy acyl CoA dehydrogenase (β-HAD)), and general oxidative capacity (marker enzyme citrate synthase (CS)).

In response to a 4 week adaptation to a high fat (~62% fat) moderate LCD (~20% CHO), skeletal muscle LPL activity increased almost 2-fold, increasing fatty acid availability to the muscle and increasing intramuscular triglyceride content significantly [30]. In terms of muscle fatty acid uptake, there is evidence that the FAT/CD36 protein and mRNA were increased modestly (1.25-fold) after only 5 d on a moderate LCD (20% CHO), while FABP_pm _gene expression and protein content were unaffected by the diet [31]. In general, muscle uptake of fatty acids and very low density lipoprotein triglycerides, as well as plasma fatty acid oxidation were higher during exercise following a fat-rich LCD (21% CHO) when exercise training was combined with the diet perturbation [32].

In human studies, skeletal muscle CPT I is unaffected by LCD. This was demonstrated at the level of maximal enzyme activity following a 6 d LCD (~3% CHO) [12], and mRNA levels following a 5 d LCD (19% CHO) [31]. However, in skeletal muscle of rats fed a high-fat diet, CPT I enzyme activity capacity was increased up to 1.3 to 2-fold at 10 weeks, depending on the fatty acid composition of the diet [33]. In another rat study, increased gene expression of CPT I mRNA appears to be restricted to type I slow oxidative muscle fibers, since a significant increase was only documented in the soleus muscle, and not the extensor digitorum longus following 8 weeks of a high-fat LCD (0% CHO) [34]. Taken together, these studies suggest that the short term 5–6 d LCD perturbation may not be prolonged enough to evoke a significant change in activity or gene expression of this enzyme which regulates transport of fatty acids into the mitochondria for oxidation. This is further supported by the fact that in well trained human subjects, maximal CPT activity was modestly increased following a prolonged (4 week) very low LCD (<20 g CHO), although it is not clear from this data whether this measurements included CPT I and CPT II activity together [35].

Increased activity of a key marker enzyme for fatty acid beta-oxidation has been observed in human skeletal muscle during prolonged LCD perturbations as well. Although a 6 d LCD (3% CHO) did not alter β-HAD activity [12], Helge et al. [36] observed increased β-HAD activity following a 4 week LCD (20% CHO) perturbation in untrained subjects. However, they found no increase in either whole body VO_2max _or CS activity, suggesting that the increase in beta-oxidation was specific rather than a generalized increase in oxidative capacity. Similarly, a more carbohydrate restricted diet (3% CHO) for 6 d did not alter CS activity in human skeletal muscle [12]. In contrast, results from some rat studies have demonstrated modest increases in CS activity of approximately 20% [37-39], with the largest increases demonstrated in type IIb fibers [40]. Although the increase in β-HAD activity in human skeletal muscle and possibly CS in rat muscle could potentially suggest an increase in oxidative capacity, recent research has demonstrated that there was no difference in human skeletal muscle mitochondrial density (as determined by electron microscopy), even though there was an increase in fat oxidation at rest and during incremental exercise following a 5 week high fat LCD (25–30% CHO) [41].

## Conclusions

In summary, following a 6 d LCD in human subjects, PDHa activation is attenuated during intense exercise and this is due at least in part to increased PDK activity and PDK4 gene expression. This decreased activation of PDHa decreases carbohydrate and increases fat oxidation during exercise. PDK activity increases in as little as 24 h on a LCD, and PDK activity increases linearly over the 6 d. Impaired glucose clearance in response to an oral glucose tolerance test was observed in healthy subjects following only 56 h of LCD, but this may be dependent on the fatty acid composition of the diet. With re-feeding of carbohydrates, PDK activity drops to pre-diet levels in 3 h, although this does not appear to correlate with mRNA concentration. If intense exercise is restricted and muscle glycogen stores and utilization rates are preserved during the LCD, the activation of the PDH complex is similar to that following a mixed diet.

The up-regulation of enzymes involved in muscle fatty acid uptake and fat oxidation appears to be slower to response to a LCD perturbation. In addition, these adaptations appear to be of a smaller magnitude. In human studies there is evidence that muscle uptake of fatty acids is up-regulated by LCD through increased maximal activity of LPL and increased FAT/CD36. However, the maximal rate of mitochondrial transport of fatty acids through CPT I appears to be resistant to adaptive changes in response to the diet. In addition, although increased maximal β-HAD activity has been documented in response to LCD, there is no evidence that the overall oxidative capacity is elevated following a LCD in human skeletal muscle.
